# Beholden: The Emotional Effects of Having Eye Contact While Breaking Social Norms

**DOI:** 10.3389/fpsyg.2021.545268

**Published:** 2021-04-26

**Authors:** Ranjit Konrad Singh, Birgit Johanna Voggeser, Anja Simone Göritz

**Affiliations:** ^1^Department of Survey Design and Methodology, GESIS – Leibniz Institute for the Social Sciences, Mannheim, Germany; ^2^Department of Psychology, Albert-Ludwigs-University of Freiburg, Freiburg, Germany

**Keywords:** embarrassment, social norms, eye contact, disinhibited behavior, laboratory experiment, insults, heart rate

## Abstract

This study looks into the role that eye contact plays in helping people to control themselves in social settings and to avoid breaking social norms. Based on previous research, it is likely that eye contact increases prosocial behavior *via* heightened self-awareness and increased interpersonal synchrony. In our study, we propose that eye contact can also support constructive social behavior by causing people to experience heightened embarrassment when they are breaking social norms. We tested this in a lab experiment (*N* = 60) in which participants read insults at the experimenter (i.e., they exhibited norm breaking behavior). In the experimental condition, participants maintained eye contact with the experimenter. In the control condition, the experimenter did not maintain eye contact. We measured embarrassment with a self-report measure, heart rate to capture arousal, and two observational indicators of embarrassment (hesitation and laughter). In line with our hypotheses, having eye contact during norm breaking behavior as compared to no eye contact led to a stronger increase in self-reported embarrassment, a higher heart rate as well as more hesitation and more laughter. We conclude that eye contact does indeed lead to more embarrassment, while breaking social norms. This implies that eye contact gives people the power to punish norm breaking in others by inducing an aversive emotional experience.

## Introduction

Across many cultures, the eyes are considered the “windows to the soul” (Emery, 2000, p. 584). Indeed, our eyes reveal much about what is going on inside us. The link between eye behavior and internal processes is so strong, that it is the basis for widely used psychometric methods. Gaze direction, for example, indicates spatial focus of outward attention and forms the basis of eye tracking methods (Krummenacher and Müller, 2020). Furthermore, pupils do not only react to light but dilate during arousal and high cognitive load (Bradley et al., 2008). Of course, that is just scratching the surface of what more complex eye movements convey about our thoughts and emotions (Emery, 2000).

That our eyes are so revealing may seem like an evolutionary disadvantage. However, humans are social animals: Cooperation is an important tool for survival. Cooperation, in turn, depends on communication, coordination, and trust. The facts that our eyes make it hard to lie, to conceal our focus of attention or hide our emotional state are advantages in the long run. This idea is the basis of the cooperative eye hypothesis, which argues that human evolution has favored communicative eyes (Tomasello et al., 2007). This line of thinking is supported by the finding that humans are unique in how clearly our gaze direction can be observed even from a distance. The reason for this is the clearly visible white sclera of human eyes. Before that high-contrast background, the direction the irises are facing is more distinguishable than with any other species (Tomasello et al., 2007).

However, that our eyes reveal so much is only one side of the coin. The other side is that eyes draw human attention, that is, humans react to others’ eyes. Even human babies already use eye information of others and follow their gaze, whereas other primates interpret head direction to infer the focus of attention and not gaze direction (Tomasello et al., 2007). In fact, humans routinely and often unconsciously use eye information to navigate the social world. Passersby predict others’ movement direction by their eyes and circumnavigate others accordingly (Nummenmaa et al., 2009).

Among all types of eye movement, one is particularly impactful: direct eye contact. Eye contact implies that someone is observing us and paying attention to an area of our bodies that conveys our inner state: the eyes. Perceiving someone’s direct gaze has been linked to many reactions, such as the draw and capture of attention, an automatic shift in experienced valence, an enhancement of self-referential memory and processing and a shift toward prosocial and less delinquent behavior (Contya et al., 2016; Hietanen, 2018). In line with the cooperative eye hypothesis, eye contact seems to be important in facilitating and maintaining constructive social interactions.

Contya et al. (2016) propose the following explanation: Being subjected to a direct gaze enhances our self-awareness. This, in turn, leads to more prosocial behavior (Contya et al., 2016). This is plausible because heightened self-awareness is conductive to increased social self-control. Vohs et al. (2008, p. 884) define self-control “as the self-exerting control to override a prepotent response with the assumption that replacing one response with another is done to attain goals and conform to standards.” In a social context, self-control means overriding maladaptive or inappropriate impulses to behave in line with moral standards, social norms, and one’s long-term goals. However, the first step of successful self-control in social interactions is that people monitor their current internal state and external situation for cues relevant to self-control (Voggeser et al., 2018). In other words, people need to pay attention to what they are thinking, feeling, and intending to do. Only then can they realize a need to regulate themselves accordingly. Thus, the role of eye contact in fostering prosocial behavior could be thought of as eye contact cueing self-awareness, self-awareness cuing social self-control, and more self-control in turn resulting in more and more successful prosocial and adaptive behavior (Carver and Scheier, 1978). However, eye contact may increase prosociality *via* other routes, as well. For example, locking eyes implies that the two people involved have synchronized (i.e., both are looking into the other’s eyes). Interpersonal synchrony “refers to instances when the movements or sensations of two or more people overlap in time and form” (Rennung and Göritz, 2016, p. 168) and as such, synchrony has been shown to enhance prosociality (Rennung and Göritz, 2016; Göritz and Rennung, 2019). It should be noted, however, that the role of synchrony in interpersonal interactions is still subject to debate. Hirsch et al. (2021), for example, found that synchrony (operationalized as cross-brain synchrony of neural activation patterns) can also occur in interpersonal disagreements.

Furthermore, research on eye contact has long assumed that emotions elicited by eye contact play a role in explaining the many effects of eye contact. However, these emotional reactions are not well understood yet (Hietanen, 2018). While cuing heightened self-awareness and the induction of synchrony both facilitate social self-control, eye contact may additionally elicit negative emotions in people who perform inappropriate social behavior. Consequently, eye contact may facilitate prosocial behavior by allowing people to punish norm breaking behavior by eliciting a negative emotional response in the norm breaker. This would mean that humans can “discipline” each other with a direct look into one another’s eyes.

The literature points toward three emotions that indicate a failure to comply with social or moral standards: Embarrassment, guilt, and shame (Tangney et al., 2007). The research on these emotions and their differences is complex, but for the purpose of this work, we summarize relevant aspects: (1) Embarrassment is an affective reaction to signals in social situations that something is amiss and that “some aspect of the self or one’s behavior needs to be carefully monitored, hidden, or changed” (Tangney et al., 2007, pp. 395). In other words, embarrassment is the reaction to a perceived public deficiency. When feeling embarrassed, people are motivated to behave in conciliatory ways to win (back) the approval of others. (2) Guilt arises from a perceived mismatch between oneself or one’s behavior with personally held moral views. A difference to embarrassment is that guilt can be experienced outside of social contexts. Stealing if there are no witnesses will likely cause guilt, but not embarrassment. Embarrassment, by contrast, can be triggered without any moral conflict, for example, by tripping in front of other people (Tangney et al., 2007). (3) Shame is not always clearly delineated from guilt. Researchers try to differentiate between shame based on different aspects, such as the type of triggering event, whether the event is public or private, and the degree to which people attribute the event to a failure of self. Concerning these emotions’ outcomes, guilt is usually seen as more adaptive, leading to constructive coping and behavioral change, whereas shame can easily lead to negative outcomes such as depression, self-esteem issues, and anxiety (Tangney et al., 2007).

Of the three emotions, this study focuses on embarrassment. Embarrassment is most related to personal concerns of how we are perceived by others. Both shame and guilt can occur even if we are not observed or do not heed the standards of others. Furthermore, unlike guilt, embarrassment does not require the person to hold the relevant social or moral standard themselves. The mere perception that others hold that standard and perceiving an own breach of that standard is sufficient to experience embarrassment. Lastly, embarrassment is a more immediate and automatic reaction than the more complex emotions guilt and shame.

Our central research question is: Does eye contact induce embarrassment in people who are breaking social norms? To answer this question, we need to bring participants into a situation where they break a social norm and then vary whether they are subject to eye contact or not. In our experiment, participants read insults out aloud at the experimenter. In one condition, the experimenter made no eye contact. In the other condition, the experimenter and participants held eye contact. With regard to the specificity of eliciting embarrassment rather than guilt or shame: Our paradigm lets participants exhibit behavior that usually breaks a moral standard. However, since participants are asked to do so by the very person who will be the target of the insults it is not an actual norm breach. What remains is an awkward, inappropriate social interaction that causes embarrassment but not shame or guilt.

As automatic emotional reactions such as embarrassment are not completely captured with self-report measures alone, we employed a three-pronged approach: self-report measures, a physiological measure, and observation measures. Specifically, we not only asked participants about their experienced embarrassment before and after voicing insults but also measured their heart rate before, during, and after voicing insults. While heart rate alone does not demonstrate embarrassment, it reflects the arousal (Kleinke and Pohlen, 1971) that accompanies a sudden negative emotion. Furthermore, we kept track of two involuntary indicators of embarrassment: Instances of laughter and instances of hesitation. To mask this study’s focus on embarrassment, we asked about several other affective states during the self-report phases, too. This experimental setup adds a new emotional perspective to eye contact research, which so far has mostly dealt with the behavior changes that result from eye contact. Our experiment, in contrast, sets the behavior as fixed and then delves into the emotional reactions.

For our experiment, we chose a lab rather than a field setting for three reasons: (1) We sought to eliminate context effects such as the communication context or established social relationships with all their dependencies. (2) We wanted to isolate immediate, non-deliberate reactions. The lab setting made it clear on a conscious level that the participants were not actually intending to insult the experimenter. Automatic, internalized reactions to dealing insults out aloud, however, would still occur. (3) It was crucial to measure non-self-report indicators of embarrassment in addition to self-reports. Neither the observational measures nor the physiological measures would have been feasible outside the lab.

In summary, our idea is that having direct eye contact, while breaking social norms is embarrassing. In terms of hypotheses, we postulate that participants having direct eye contact, while reading insults to an experimenter as compared to participants not having eye contact (H1) have a higher heart rate, (H2) report higher increases of embarrassment, (H3) display more behaviors indicative of embarrassment (i.e., laughing or hesitating before reading out an insult), and (H4) experience a stronger worsening of their mood.

## Materials and Methods

### Ethics Statement

We conducted this study in accordance with the APA ethical standards and the German Psychological Society’s (DGPs) ethical guidelines (DGPs, 2004, C.III). According to the DGPs ethical guidelines, an institutional research board’s ethical approval is only required if funding is subject to such an ethical review. No such requirements were present for this study. All participants were of legal age. Participation in the study was voluntary, no monetary reward was granted. Participants were given the option to participate in a raffle for a cinema ticket and the choice to pick a treat after completing the study. In the case of students at the psychology department of the University of Freiburg, they could also receive course credit of 0.5 h. All participants were told beforehand that the task they would be asked to perform in the course of the study contained taboo words. Participants gave informed consent to this as well as to the usage of their data upon entering the study. Furthermore, participants were made aware that they could abort the study at any time without repercussions. In case of emotional distress, participants were given the chance to talk and recover and only left the session when they reported adequate emotional wellbeing. All data were collected and analyzed anonymously. The equipment used to measure heart rate was sanitized before and after each session.

### Sample

We recruited participants *via* flyers and *via* the participant pool of the department of psychology of the University of Freiburg. A total of 68 persons participated in the experiment, most of them students. Initial technical difficulties prevented the heart rate measurement of four participants; hence the final sample was *N* = 64. The sample consisted of 50 women (78%) and 14 men (22%). On average, the participants were 22 years old (*SD* = 3). Participants were randomly assigned to the eye contact condition (*n* = 32) or to the no eye contact condition (*n* = 32).

### Design and Procedure

This mixed-design experiment consisted of three parts: (1) a computer-administered questionnaire, (2) reading aloud insults with the manipulation of eye contact vs. no eye contact resulting in two experimental conditions, and (3) another computer-administered questionnaire. Both questionnaires were identical for all participants, regardless of experimental condition.

After giving consent, participants were equipped with a chest belt to measure their heart rate and sat opposite of the experimenter at a desk. The same experimenter conducted all sessions. Next, participants were randomly assigned to either the eye contact or the no eye contact condition by drawing lots. The experimenter read a standardized text detailing the procedure. Following this, participants filled out the first questionnaire. The first page informed about the anonymized collection and analysis of the data for scientific purposes as well as the possibility to withdraw from the experiment at any time without repercussions. Participants gave informed consent by clicking the button prompting the next page. The demographics asked for age, sex, and highest educational attainment. Additionally, participants were asked about their physical fitness, regular physical activity, impairments (e.g., asthma), as well as any preceding or unusual physical exertion (e.g., running up the stairs to the lab). Next, participants reported their current emotional state twofold, using (1) part of the self-assessment manikin (SAM; Bradley and Lang, 1994) and (2) an adapted, shortened version of the Eigenschaftswörterliste (attribute word list, EWL; Janke and Debus, 1978). On the last page, participants received the instructions to inform the experimenter that they had finished. Starting the second part, the experimenter read another standardized text explaining the experimental task to the participants: They had to read a list of 50 insults out loud, looking at the experimenter, while speaking each insult. In the eye contact condition, the experimenter held eye contact with the participant, while the insults were spoken. In the no eye contact condition, the experimenter kept her gaze on her notes. The experimenter’s posture was otherwise kept identical, and the distance between participant and experimenter was kept at or below 80 cm during the experiment. Any comprehension questions were answered before the task began. During the task, participants spoke the 50 insults in randomized sets of five consecutive words, followed by a short pause, while they picked up the next set. This made sure no participant could speed through the task before heart rate measurement could be taken. The frequency of certain behaviors was observed and noted down, namely how many times participants snickered, giggled, or laughed, and if they hesitated before speaking one of the insults out loud.

After finishing the 50 insults, participants were instructed to fill out the second questionnaire on the laptop, which again contained the two measurements for participants’ current emotional state, including embarrassment. Lastly, participants were given the chance of providing feedback. Each experimental session was planned with a buffer of 15 min to allow participants to talk about the experiment and relax. Any potentially remaining emotional distress was discussed and alleviated at this point, and the participants were sent off with a treat of their choice once they reported adequate wellbeing and also appeared to be in a reasonably good mood.

### Materials

#### Insults

The 50 insults were taken from instances of flaming on the Internet. We sought to avoid politically charged insults. As such, we did not use racist and homophobic slurs and kept sexism to a minimum (four of the 50 insults can be conceived as sexist – two misandrist and two misogynist). The 50 insults were printed on laminated cards, which were randomized for each participant and presented in sets of five.

#### Self-Assessment Manikin: Mood and Arousal

The SAM is a pictorial assessment technique that measures participants’ affective reaction to stimuli (Bradley and Lang, 1994). We chose the SAM as an economical and easy-to-understand measure. We presented two rows of pictures: One measuring the current mood valence and one measuring arousal. Regarding valence, participants indicated how they felt by choosing an abstract representation of a person who is frowning deeply, frowning slightly, looking neutral, smiling slightly, or smiling broadly. We added the scale anchors “unhappy” and “happy” to the two outmost pictograms to make it self-explanatory. Regarding arousal, participants indicated how they felt by choosing an abstract representation of a person with a neutral, straight mouth position. Arousal was indicated 3-fold. The eyes changed from closed to wide open. The eyebrows raised gradually. A dot in the lowest arousal gradually transformed into a star shape with squiggly lines indicating restless vibration in the highest arousal condition. We added the scale anchors “relaxed” and “excited” to the two outmost pictograms to make it self-explanatory.

#### Embarrassment Self-Report Measure

We assessed the change in participants’ level of embarrassment caused by the task by directly asking them how embarrassed they felt right now before the task and afterward. To not alert the participants to our interest in embarrassment, we used a selection of items from the EWL (Eigenschaftswörterliste “List of attribute words”; Janke and Debus, 1978) as distractors. The EWL is a scale measuring a person’s state in 15 different emotions with 161 items in total. In our study, we used one item from each subscale. In total, we used 16 statements about the current emotional state (e.g., “I am feeling angry”). Of those, 15 were derived from the 15 different emotional categories of the EWL. The 16th, our focus item “I am feeling embarrassed,” was added for our study and is not part of EWL. Each statement was answered on a five-point Likert scale: “Does not apply at all,” “Does not apply,” “Partially applies,” “Applies,” and “Strongly applies.”

#### Heart Rate Measurement

As a measure of arousal, we measured heart rate using a Beurer PM25 heart rate monitor watch and chest-belt. In total, 13 heart rate measurements were taken from every participant. Two were taken before the insult task: the first after outfitting the participant with the heart rate belt and the second after they filled out the first part of the questionnaire. During the task, 10 measurements were taken; one after each set of five insults. The last measurement was taken after the participants had finished filling out the second questionnaire.

#### Observation Measure: Hesitation and Laughter

As a behavioral indicator, instances of hesitation as well as laughter were counted by the experimenter. The resulting score reflects the number of occurrences during the experiment. The process of noting down instances was not visible to the participants.

#### Software for Data Analysis

Analyses were performed with R (R Core Team, 2018) within RStudio (RStudio Team, 2020). To import and wrangle our data, we used the packages tidyverse (Wickham, 2017), readxl (Wickham and Bryan, 2018), haven (Wickham and Miller, 2018), and sjlabelled (Lüdecke, 2018). For statistical analyses, we used the packages car (Fox and Weisberg, 2011) and nlme (Pinheiro et al., 2018). To create result reports and plots, we used knitr (Xie, 2018), kableExtra (Zhu, 2018), Cairo (Urbanek and Horner, 2015), and ggplot2 (Wickham, 2016).

## Results

To assess if eye contact influenced embarrassment, while reading insults at the experimenter, we analyzed participants heart rate, the observational variables (i.e., instances of laughter and instances of hesitation), as well as changes in self-reported embarrassment before and after reading the insults out loud. We also looked into self-reported arousal. Lastly, we provide an exploratory analysis on the effects of the manipulation on other emotional states. Note, however, that we included these other emotions as distractors and provide the results as inspiration for future research and in the spirit of transparency.

### Heart Rate

**Figure 1:**
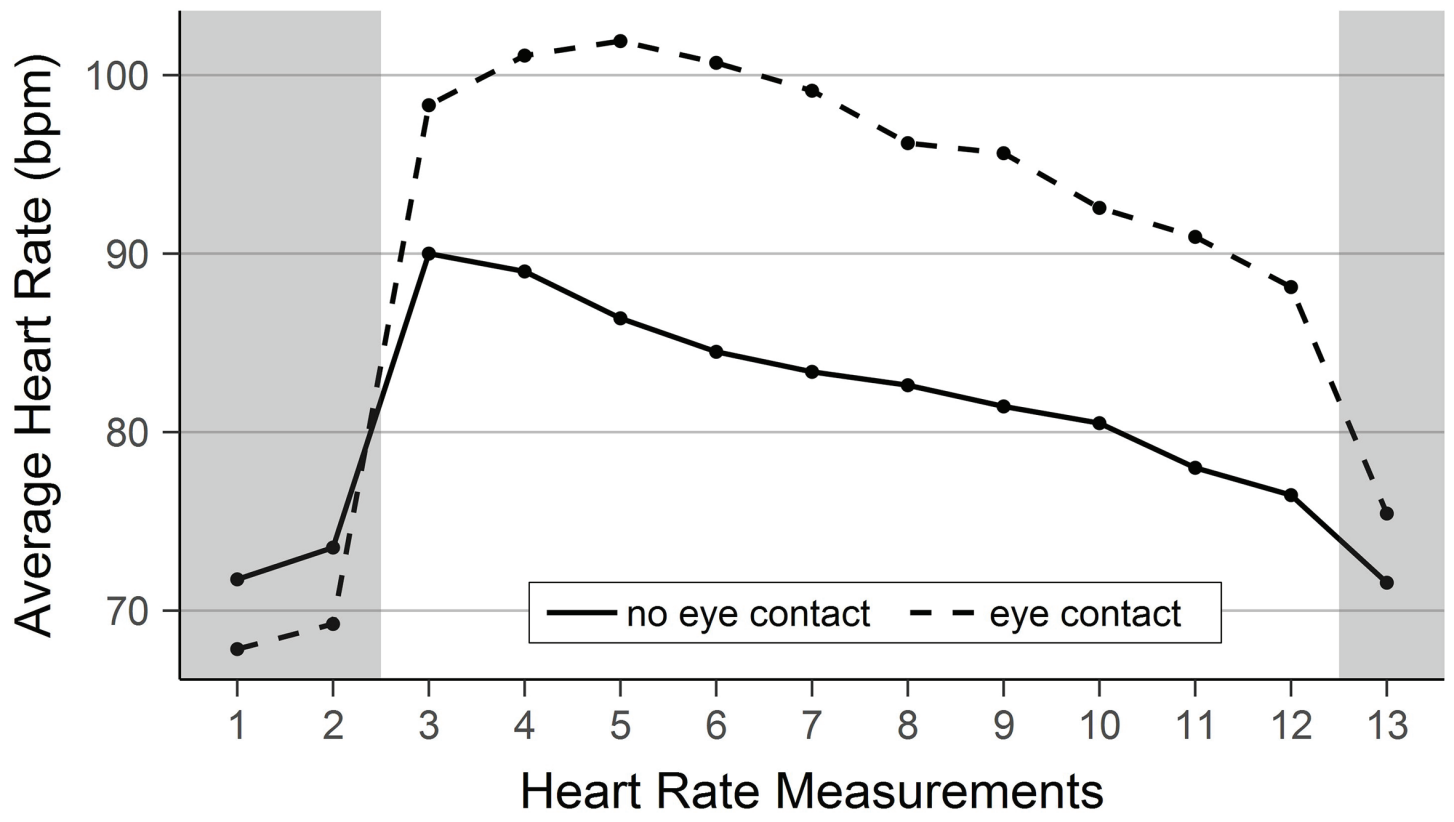
Average heart rate in the two conditions over time. The figure shows the average heart rate measured at 13 points during the experiment. The two conditions are displayed separately. Please note that only the points are measurement instances. The lines were added for better visibility.

Figure 1 shows participants’ average heart rate during the experiment. The gray areas signify the time before and after reading the insults out loud. Specifically, measurements 1, 2, and 13 were taken before and after speaking the insults, while measurements 3–12 were taken during voicing insults. Descriptively, we see (1) a heart rate increase as the insulting begins, (2) a slow decrease of heart rate during the insulting, and (3) markedly higher heart rates throughout the insulting in the eye contact condition when compared with the no eye contact condition.

**Figure 2:**
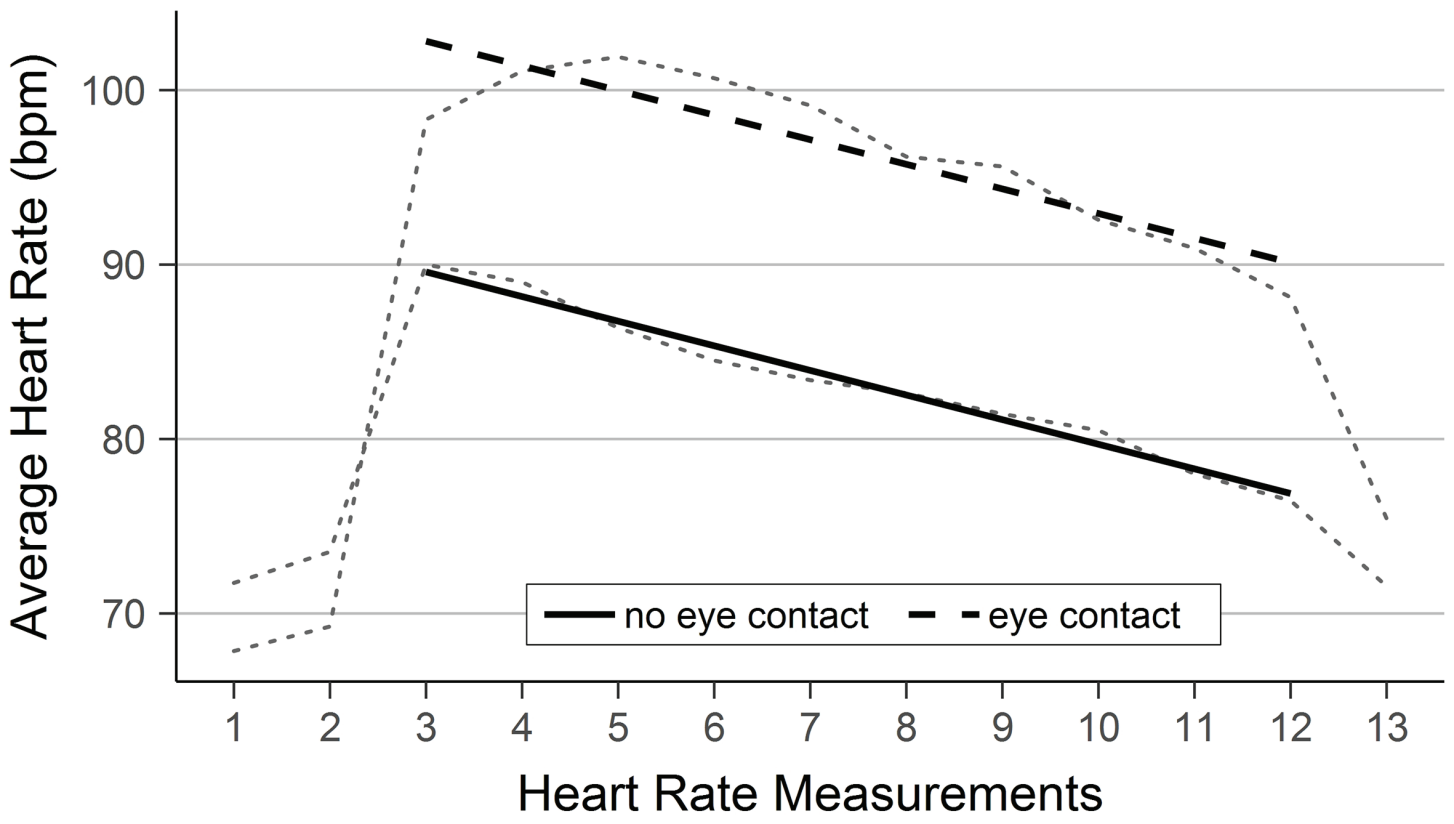
Fitted regression lines of heart rate in the two conditions over time. The figure shows the heart rate predicted by the regression. The bold, solid line shows the prediction for the no eye contact condition and the bold, dashed line shows the prediction for the eye contact condition. The model includes all measurements taken during the reading of insults (three through 12). The light, dotted lines in the background show the observed development of the average heart rates (cf. Figure 1).

To perform inferential tests, we set out by fitting an appropriate model. Because our hypothesis pertains to the effect of condition on the heart rate during the insulting, we only include the relevant heart rate measurements (i.e., 3–12) in the models. We first tested if a multilevel model is appropriate to capture the repeated heart rate measurements. To this end, we compared an intercept-only OLS regression with an intercept-only multilevel model in which the intercepts varied. Adding random intercepts led to a better model fit, *Χ*^2^(1) = 730.84, *p* < 0.001. This means that participants vary in their average heart rate, and random intercepts capture this variability. We then built up the model by first adding condition as a predictor, *Χ*^2^(1) = 21.63, *p* < 0.001, and next the measurement point as a predictor, *Χ*^2^(1) = 322.32, *p* < 0.001. Both significantly increased explanatory power. We then tested if this multilevel model (i.e., condition and measurement point as predictors and varying intercepts for participants) could be improved by adding random slopes. However, letting slopes vary between the two conditions did not improve model fit, *Χ*^2^(2) = 0.01, *p* = 0.993. In other words: The effect of eye contact on the heart rate did not vary across participants. Consequently, we retained the random intercept model. Lastly, we tested if adding the interaction between condition and measurement point improved the model. It did not, *Χ*^2^(1) = 0.22, *p* < 0.640, implying that the effect of measurement point did not vary between the two conditions. In summary, the best model lets the intercept (i.e., the average heart rate) vary across participants and includes condition as well as measurement point as predictors without an interaction between them (*−2LL* = −2051.25). To include the condition as a predictor, we use indicator coding with the no eye contact condition as the reference category (i.e., no eye contact was coded as 0, eye contact as 1). Heart rate was significantly higher in the eye contact condition as compared to the no eye contact condition, *B* = 13.23, *t*(62) = 5.06, *p* < 0.001. In the course of reading insults out loud, the heart rate decreased from measurement point to measurement point, *B* = −1.41, *t*(575) = −20.74, *p* < 0.001. The rate of the decrease was not significantly different in the two conditions, as shown earlier (i.e., interaction between condition and measurement point did not significantly increase explanatory power). Figure 2 shows the mean heart rate (dotted lines) and the fitted regression lines for the two conditions.

This result supports our hypothesis that eye contact during norm breaking behavior leads to a higher heart rate, indicating higher arousal. Visually, this can be seen in the dashed regression line (eye contact condition) being in higher heart rate ranges than the solid regression line (no eye contact condition). We also found that the heart rate gradually decreases as more and more insults had been spoken and participants got used to the situation. Still, this decrease has the same rate in both conditions, meaning that the effect of eye contact on the heart rate remained stable during the entire phase of reading insults out loud. This is visible in the negative slope of both regression lines (decrease over time) and that the two lines are parallel (i.e., the rate of decrease over time is the same in both conditions).

### Observational Variables

We analyzed the two observational measures of embarrassment (Table 1): (1) how often did participants laugh during the insulting, and (2) how often participants hesitated during the insulting. Please note that the distribution of the number of instances had zero as its lower bound. Due to the resulting floor effect, the measures have unequal variances between the two conditions. We consequently compared the number of occurrences using Welch’s *t*-test for unequal variances. Participants laughed more often in the eye contact condition (*M* = 6.34) than in the no eye contact condition (*M* = 3.56), *t*(54.53) = −3.79, *p* < 0.001. Moreover, participants hesitated more often in the eye contact condition (*M* = 3.06) than in the no eye contact condition (*M* = 0.84), *t*(49.95) = −5.56, *p* < 0.001.

**Table 1:** Mean and SD for observational and self-report outcome measures by condition.

Time of measurement	Measure	no eye contact mean (SD)	eye contact mean (SD)
Before reading insults (Baseline)	Self-reported embarrassment	1.44 (0.76)	1.47 (0.62)
	SAM positive mood	3.75 (0.72)	3.88 (0.71)
	SAM arousal	2.50 (0.92)	2.34 (0.83)
During reading insults	Instances of laughter	3.56 (2.33)	6.34 (3.43)
	Instances of hesitation	0.84 (1.14)	3.06 (1.95)
After reading insults	Self-reported embarrassment	2.88 (1.26)	3.56 (1.13)
	SAM positive mood	3.22 (0.97)	2.91 (0.96)
	SAM arousal	3.19 (1.09)	3.41 (0.80)

### Self-Reported Mood and Embarrassment

Experienced embarrassment, self-reported valence, and self-reported arousal were measured before and after voicing insults (Table 1). Consequently, condition is a between-subject factor and time of measurement (pre vs. post) is a within-subject factor. We use multilevel models to capture this data structure. Intercepts are allowed to vary across participants, accounting for initial differences between participants. The models include condition (no eye contact as a reference), the pre-post factor (pre as a reference) and the condition by pre-post interaction as predictors. Since the two factors are indicator coded, the three coefficients signify the following: (1) The condition coefficient is the mean difference between the two groups before the insulting. (2) The pre-post coefficient is the mean difference between the measurement after the insulting and measurement before the insulting in the no eye contact condition. (3) The interaction is the mean difference between the eye contact condition after the insulting and the no eye contact condition after the insulting. With this design, an effect of the manipulation (i.e., eye contact) would show by a significant interaction effect. Before the insulting, embarrassment did not differ between conditions, *B* = −0.03, *t*(62) = 0.13, *p* = 0.899. The insulting increased embarrassment in the no eye contact condition, *B* = 1.44, *t*(62) = 6.32, *p* < 0.001. The insulting lead to a significantly greater increase in the eye contact condition than in the no eye contract condition, *B* = 0.66, *t*(62) = 2.04, *p* = 0.046. Please keep in mind that the B pertaining to the last effect is smaller, because it signifies the added effect in the eye contact condition as compared to the no eye contact condition. The overall change between before and after reading insults out loud is *B* = 1.44 + 0.66 = 2.1. That means eye contact increased the effect of reading insults on experienced embarrassment. Regarding valence, participants’ mood before the insulting did not differ between the conditions, *B* = 0.13, *t*(62) = 0.89, *p* < 0.559. Speaking insults deteriorated participants mood in the no eye contact condition, *B* = −0.53, *t*(62) = −3.38, *p* = 0.001. Speaking insults also deteriorated participants’ mood somewhat more in the eye contact condition than in the no eye contact condition, but this difference narrowly failed a conventional level of significance, *B* = −0.44, *t*(62) = −1.97, *p* = 0.053. Regarding self-reported arousal, we found no difference between the two conditions before the insulting, *B* = −0.16, *t*(62) = −0,68, *p* = 0.497. The insulting increased arousal in both conditions, *B* = 0.69, *t*(62) = 3.81, *p* < 0.001. In the eye contact condition, arousal increased somewhat more than in the no eye contact condition, but this difference failed a conventional level of significance, *B* = 0.38, *t*(62) = 1.47, *p* = 0.147. In summary, speaking insults at the experimenter lead to more self-reported embarrassment, worse self-reported valence of mood, and increased self-reported arousal. Having to look the experimenter in the eye, while speaking insults, however, lead to even more self-reported embarrassment.

Lastly, we looked into the remaining emotional variables that were included as distractors to conceal the interest in embarrassment. These analyses are exploratory in nature, because we postulated no hypotheses beforehand. Since these variables were measured pre and post reading insults aloud, just like valence, arousal, and embarrassment, we analyzed them in the same way. Table 2 gives the B coefficients and marks significant effects. In summary, no emotion varied between the conditions before the insulting. Reading insults aloud reduced participants’ self-confidence, and made them angrier and feel sillier regardless of condition. However, participants’ feeling of being withdrawn and sad increased more in the eye contact condition than in the no eye contact condition.

**Table 2:** Self-reported emotions: Differences between conditions and over time.

Emotion	Difference between eye contact and no eye contact before speaking insults	Difference between pre and post speaking insults in the no eye contact condition	Difference between eye contact and no eye contact post speaking insults
Absentminded	−0.06	−0.19	0.19
Angry	0.16	0.56^***^	−0.16
Energetic	0.25	−0.25	−0.41
Excited	−0.03	0.25	0.41
Fearful	0.03	0.25	0.00
Focused	0.06	−0.13	−0.28
Happy	0.09	−0.34	−0.53
Lethargic	−0.03	0.03	−0.22
Sad	−0.09	0.19	0.53^*^
Self-confident	0.00	−0.66^***^	0.06
Sensitive	0.09	0.28	0.25
Silly	0.09	1.28^***^	−0.31
Talkative	0.00	−0.28	0.03
Tired	−0.09	0.13	−0.03
Withdrawn	−0.19	0.13	0.63^**^

For each emotion, a separate multilevel model was calculated.

* *p* < 0.05;

** *p* < 0.01;

*** *p* < 0.001.

Values refer to a scale from 1 to 5.

## Discussion

The goal of this study was to examine the emotional effects of eye contact on people who break social norms. Specifically, we tested if eye contact during norm breaking behavior led to more embarrassment. As norm breaking behavior, participants spoke insults to the experimenter. In one condition, the experimenter kept her gaze on her notes, while in the other condition, the experimenter maintained eye contact, and participants were asked to do the same. We assessed embarrassment with three types of measures: A physiological measure of the accompanying arousal (i.e., heart rate), two observational measures (i.e., instances of laughter and instances of hesitation) during speaking the insults and self-report measures (i.e., experienced embarrassment and mood) before and after speaking insults. Our results show that eye contact significantly increased embarrassment in all three types of measures: Eye contact lead to more arousal (as measured by heart rate), more laughter and hesitation, and higher self-reported embarrassment. The SAM measures for arousal and mood fell short of being conventionally significant but showed a tendency toward higher arousal and worse mood. These findings support that eye contact causes negative emotions in people who behave inappropriately. Emotions, and especially embarrassment, seem to be a promising piece in the puzzle of how eye contact lets people behave socially adjusted.

Our study does not offer an alternative explanation of how eye contact facilitates self-control in social settings. The mechanisms of increased self-awareness (Contya et al., 2016) and synchrony (Rennung and Göritz, 2016; Göritz and Rennung, 2019) and even other yet undiscovered mechanisms may work in parallel or in complex interaction with one another. However, our findings imply that there is an additional emotional pathway or emotional component to how socially adjusted behavior is brought about. That eye contact during norm-breaking behavior elicits feelings of embarrassment entails that humans can restrain or punish others by looking them in the eye. This powerful tool underlines the importance of researching the emotional effects of eye contact (Hietanen, 2018). Furthermore, our study demonstrates the automaticity of the embarrassment response: Although participants were aware that their norm breaking behavior was expected and even desirable in the setting of the study, the norm breaking behavior triggered embarrassment, and eye contact increased the embarrassment. A difference between the embarrassment pathway and the proposed mechanisms of self-awareness and synchrony is that embarrassment specifically pertains to eye contact in the context of inappropriate behavior, while the other two explanatory approaches are not specific to this context.

The present study is subject to some limitations. Chief among them is that we were unable to include an additional condition with eye contact but reading neutral words instead of insults. This condition would have helped to isolate the effect of eye contact itself (i.e., while not breaking social norms). However, participants and the experimenter interacted normally with each other including making eye contact before and after the insult reading phase. During these phases, the heart rate was markedly lower than during the insult reading phase (measurements 1, 2, and 13 vs. measurements 3 through 12). Consequently, we think it unlikely that mere neutral eye contact would lead to such a marked increase in participants heart rate. Nonetheless, an additional condition with neutral words should be strongly considered for future research on the topic.

Given the aim to examine whether eye contact when breaking a social norm increases embarrassment, our choice of a lab-based study as a controlled setting seems appropriate. The experiment at hand underlines the usefulness to complement self-report measures with physiological and observational measures. The heart rate measurement and the observational measures of embarrassment reveal a clear effect that eye contact increases arousal and signs of embarrassment more than no eye contact. Meanwhile, self-report measures of emotion, arousal, and mood had lower power. In case of the simple SAM self-report measure the findings even dropped below a conventional level of significance. Future research should also consider using more finely grained measures than the five-point SAM scales.

Regarding physiological measures, future research may consider alternatives to our heart rate measure. The heart rate was and is considered to reflect arousal (Kleinke and Pohlen, 1971). However, the literature there is a shift toward looking at the heart rate variability instead of absolute heart rate values. And heart rate variability does indeed seem to reflect arousal well (e.g., Choi et al., 2017). Furthermore, measures of pupil dilation (Bradley et al., 2008) or skin conductance (Rosebrock et al., 2017) could also be considered for future.

Future research should also attempt to test these findings in more versatile samples. Our sample is rather small, well educated, and predominantly female. This is acceptable to explore the effect in principle, but to gauge the size of the emotional impact in practice, a larger sample size and a more diverse sample composition are desirable. Samples with a more balanced sex (or gender) ratio may also help look into whether sex (or gender) moderates the effects we found.

We had one experimenter conduct all sessions of the experiment. It cannot be precluded that this might have caused experimenter effects. However, all actions to be performed and notes to be taken by the experimenter were extensively practiced beforehand; moreover, there was a high degree of standardization in dealing with participants, for example, the instructions were read from a sheet, and the questionnaires were self-administered *via* a laptop. Furthermore, the experimenter was not involved in preparing and writing this manuscript. We deliberately chose one experimenter to eliminate the effect of experimenter look, voice, sex, height, etc. Since this was a study run in a psychology department, we expected a majority of female participants; that is why, we chose a female experimenter with an approachable demeanor. Furthermore, the experimenter is a trained nurse practiced in the use of heart rate measurement equipment and had the necessary skills and knowledge to calm and reassure participants who might have been affected by the task.

It should also not be overlooked that the way eye contact is interpreted may vary between different cultures. Akechi et al. (2013), for example, found that people from an East Asian country (specifically Japan) interpreted faces making eye contact as angrier, more unapproachable and more unpleasant than participants from a Western European country (specifically Finland). We would still expect eye contact to be effective in activating self-awareness and self-control. However, its impact may be stronger and have a more negative emotional connotation in East Asian countries. International replications of the experiment ware consequently desirable.

Despite its limitations, our study points the way to a new perspective on social self-regulation and the role of eye contact. As such, the new perspective promises interesting implications for applied research fields. In clinical research on social anxiety, for example, it is a long standing finding that socially anxious people fear eye contact (Schneier et al., 2011). The link between eye contact and embarrassment our study demonstrates may be a helpful puzzle piece to better understand the processes underlying social anxiety: Eye contact can make people experience (more) embarrassment when they show behavior they feel is seen as inappropriate. This may contribute to socially anxious people fearing eye contact because socially anxious people may (1) experience eye contact induced embarrassment in more situations than less socially anxious people, (2) experience eye contact induced embarrassment more strongly, and/or (3) fear anticipated eye contact induced embarrassment more strongly. Our findings may inspire new avenues for research. Furthermore, our study suggests that eye contact during awkward situations increases embarrassment automatically, even though people are aware that they behave as expected. This shows the challenge that it is not enough to cognitively understand that a behavior is acceptable to avoid mortification.

Eye contact and social self-regulation is also of interest to researchers dealing with disinhibited social behavior in online settings. Often referred to as online disinhibition (OD), this antisocial communication behavior ranges from spontaneous flaming and hate speech to pre-mediated cyber-bullying with potentially lethal consequences for the victims (Voggeser et al., 2018). OD creates immense challenges for online platforms (e.g., Wong, 2019). Early research had focused on anonymity as the likely cause of OD. However, OD occurs in non-anonymous settings, as well (Rainie et al., 2012). Other research has thus compared the effect of anonymity to other aspects of online communication (Lapidot-Lefler and Barak, 2012). What emerged was that a lack of eye contact between communication partners was the strongest predictor of OD. The absence of this pathway of social influence seems crucial to understand antisocial online behavior. The findings of our study may be transferred to an online context and suggest that embarrassment likely plays a role in social behavior regulation. This opens up the way for future research on how embarrassment-inducing social sanctions (e.g., implementing or simulating eye contact in an online setting) can work online to combat OD.

Whether in basic or applied research, looking further into the role that embarrassment plays in eye contact seems promising. In the end, it seems that while looks can only kill proverbially, they can certainly mortify.

## Data Availability Statement

The raw data supporting the conclusions of this article will be made available by the authors, without undue reservation.

## Ethics Statement

Ethical review and approval was not required for the study on human participants in accordance with the local legislation and institutional requirements. The patients/participants provided their written informed consent to participate in this study.

## Author Contributions

RS: conception of the work, analysis and interpretation of data, major drafting, revising, and final approval, and agrees to be accountable for all aspects of the work. BV: conception and design of the work, acquisition and interpretation of data, drafting, revising, and final approval, and agrees to be accountable for all aspects of the work. AG: conception of the work, revising and final approval, and agrees to be accountable for all aspects of the work. All authors contributed to the article and approved the submitted version.

### Conflict of Interest

The authors declare that the research was conducted in the absence of any commercial or financial relationships that could be construed as a potential conflict of interest.
